# Acute cardiac side effects after COVID-19 mRNA vaccination: a case series

**DOI:** 10.1186/s40001-022-00695-y

**Published:** 2022-06-02

**Authors:** Noemi F. Freise, Milena Kivel, Olaf Grebe, Christian Meyer, Bahram Wafaisade, Matthias Peiper, Tobias Zeus, Jan Schmidt, Judith Neuwahl, Danny Jazmati, Tom Luedde, Edwin Bölke, Torsten Feldt, Björn Erik Ole Jensen, Johannes Bode, Verena Keitel, Jan Haussmann, Balint Tamaskovics, Wilfried Budach, Johannes C. Fischer, Wolfram Trudo Knoefel, Marion Schneider, Peter Arne Gerber, Alessia Pedoto, Dieter Häussinger, Martijn van Griensven, Amir Rezazadeh, Yechan Flaig, Julian Kirchner, Gerald Antoch, Hubert Schelzig, Christiane Matuschek

**Affiliations:** 1 Department of Gastroenterology, Hepatology and Infectious Diseases, University Hospital Düsseldorf, Medical Faculty, Heinrich Heine University, Düsseldorf, Germany; 2 Department of Pediatric, University Hospital Düsseldorf, Medical Faculty, Heinrich-Heine-University, 40225 Düsseldorf, Germany; 3 Department of Cardiology and Rhythmology, Petrus Hospital, Wuppertal, Germany; 4 Department for Cardiology, Rhythmology, Angiology and Intensive Care Medicine, cNEP Research Consortium, EVK Düsseldorf, Düsseldorf, Germany; 5 Department of Radiation Oncology, University Hospital, Medical Faculty, Heinrich Heine University, Moorenstr. 5, 40225 Düsseldorf, Germany; 6 Medical Faculty, Department of Cardiology, University Hospital of Düsseldorf, Düsseldorf, Germany; 7 Department of Gastroenterology, Hepatology and Infectious Diseases, University Hospital Magdeburg, Medical Faculty of Otto Von Guericke University Magdeburg, Leipziger Str. 44, 39104 Magdeburg, Germany; 8 Institute for Transplant Diagnostics and Cell Therapeutics, Heinrich Heine University, Düsseldorf, Germany; 9 Medical Faculty, Department of Surgery and Interdisciplinary Surgical Intensive Care Unit, Heinrich Heine University, Düsseldorf, Germany; 10 Division of Experimental Anesthesiology, University Hospital Ulm, Ulm, Germany; 11 Medical Faculty, Heinrich-Heine-University, 40225 Düsseldorf, Germany; 12 Department of Anesthesiology, Memorial Sloan Kettering Cancer Center, NY, USA; 13 Department cBITE, Maastricht University, MERLN Institute for Technology-Inspired Regenerative Medicine, Maastricht, The Netherlands; 14 Medical Faculty, Department of Diagnostic and Interventional Radiology, University Hospital of Düsseldorf, Dusseldorf, Germany; 15 Medical Faculty, Department of Vascular Surgery, University Hospital Heinrich Heine University, Düsseldorf, Germany

**Keywords:** Inflammation, COVID-19, Myocarditis, mRNA vaccine

## Abstract

**Background:**

Vaccination against SARS-CoV-2 has been the main tool to contain the pandemic. The rush development of the 3 vaccines and their expedited approval have led to inoculation of millions of patients around the world, leading to a containment of the disease. Despite continuous viral mutations and the identification of weaker variants, the severity of the infections has been mild, with many patients being either asymptomatic or recovering at home. Currently the focus has shifted from the host of organ damage related to the infection to potential side effects of the vaccine. Myocarditis has been reported as one of the potential side effects from the mRNA vaccine, affecting young healthy individuals.

Up to September 30, 2021, 1.243 cases of myocarditis after vaccination with BNT162b2 Comirnaty© were registered in young adults by the Paul-Ehrlich-Institute in Germany alone. The exact pathophysiology and the risk factors for myocarditis following vaccination remain unclear. We present a case series of eight patients with cardiac symptom shortly after SARS-CoV-2 mRNA vaccination (BNT162b6, Biontech, Comirnaty© or mRNA-1237 Moderna, Spikevax©).

**Patients and methods:**

Eight patients between 13 and 56 years of age, vaccinated with either BNT162b2 or mRNA-1273 mRNA vaccine between January and August 2021 developed cardiac side effects shortly after either their first or second dose of the vaccine. Clinical data were retrieved from the clinical information system and analyzed. To support diagnosis of myocarditis or pericarditis, cardiac magnetic resonance imaging (MRI) was performed shortly after the onset of symptoms, with further investigations in severe cases. Symptoms were defined as dyspnea, chest pain and cardiac arrhythmia as determined by electrocardiography.

**Results:**

Eight patients (5 males and 3 females) developed cardiac symptoms compatible with myocarditis, according to the CDC criteria, shortly after SARS-CoV-2 mRNA vaccination. Three patients (2 males, 1 female) required hospitalization due to severe chest pain and elevated troponin levels. All patients recovered fully within 7 days from the symptom onset.

**Conclusions:**

Our data suggest that cardiac adverse events such as myocarditis or pericarditis shortly after SARS-CoV-2 mRNA vaccination are rare but possible and occur particularly in male patients.

## Introduction

Since the first report of the coronavirus SARS-CoV-2 infection, there has been no major treatment identified the virus. Most of the proposed therapy is supportive to alleviate the symptoms. The main focus of the past 2 years has been on vaccination.

The first mRNA-based vaccines targeting SARS-CoV-2 were developed by Biontech Pfizer (BNT162b2, Comirnaty©) and Moderna (mRNA-1273, Spikevax©), investigated by large prospective randomized trials and made available to the world at an extraordinary speed. In multiple studies, mRNA-based vaccination significantly reduced the rate of infection and transmission of SARS-CoV-2, resulting in fewer hospitalizations and deaths [1–4] After gaining approval, a worldwide vaccination campaign started at the end of 2020. After 1 year of use, vaccine related side effects started to be reported. Due to the high number of vaccinations administered in a short period, complications apparently occurred more frequently when compared with a standard flu vaccination. In addition, adverse events due to vaccination are currently receiving a large coverage from the media, questioning their safety.

**Fig. 1:**
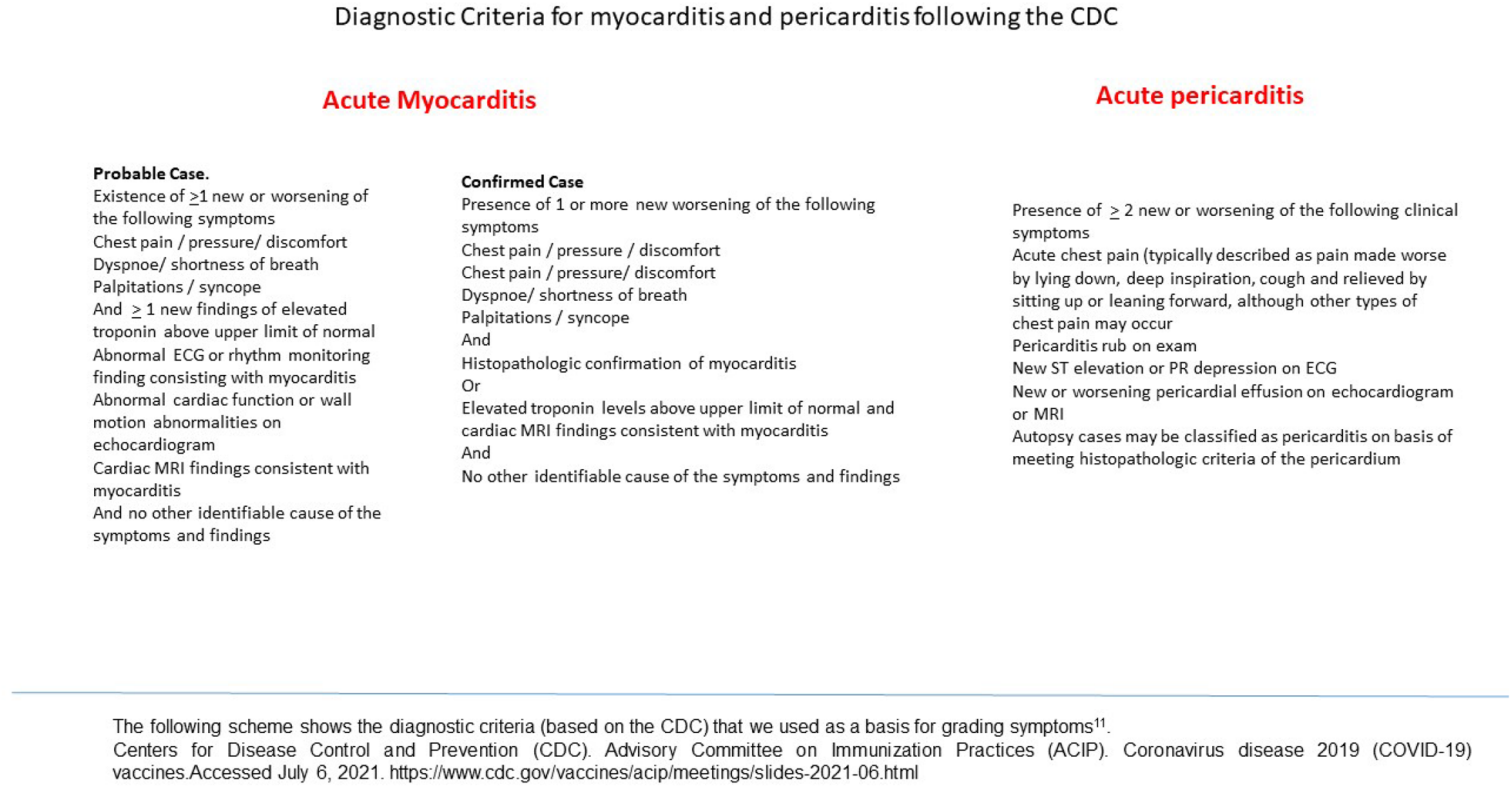
Diagnostic criteria for myocarditis and pericarditis following the CDC

The gold standard for the diagnosis of myocarditis is still a myocardial biopsy, a quite invasive test associated with potential serious complications, which is not routinely performed [5, 6]. In the presence of high index of suspicion, diagnostic criteria for myocarditis or pericarditis following the Center of Disease Control  and Prevention (CDC) (Fig. 1) are used. This is a rare disease, affecting approximately 10–20 per 100,000 population [7], responsible for 10% of cases of sudden death in the young patients, as shown in autopsy-based series [8]. Myocarditis is often triggered by viral infections or caused by post-viral immune-mediated responses, leading in 30% of the cases to dilatative cardiomyopathy [9].

Post-vaccination myocarditis has been reported for the first time in 1957 by Dalgaard et al. after vaccination against smallpox [10]. Now it is described as a serious complication following mRNA-based vaccines with BNT162b2 or mRNA-1273, mostly affecting young healthy adults [11–34]. This is particularly important, since this subgroup of individuals is not at high risk for severe COVID-19 disease course. Since the two vaccines are substantially different, further studies on the pathophysiology of their side effects are of high interest, especially to improve acceptance of the vaccines among the population.

Here, we report a case series of eight consecutive patients with cardiac side effects after inoculation of either the first or the second dose of a mRNA vaccine against SARS-CoV-2.

## Patients, material and methods

This is a retrospective analysis of 8 consecutive patients (5 males, 3 females) who presented to our institution with symptoms compatible with myo- or pericarditis shortly after first or second dose of the SARS-CoV-2-mRNA-vaccine. Vaccination was performed with either BNT162b6 or mRNA-1273, at the recommended time intervals. Interpretations of the presented symptoms and determination of the diagnosis of myocarditis or pericarditis was based on the diagnostic criterias of the Center of Disease Control and Prevention (CDC) (Fig. 1). Medical history and further diagnostic tests, e.g., coronary catheterization, excluded other causes. The reports of cardiac side effects were taken from the Departments of Infections Disease at the University of Dusseldorf, Department of Pediatrics University of Dusseldorf, Department of Cardiology University of Dusseldorf, Department of Cardiology and Rhythmology, Petrus Hospital, Wuppertal, Department for Cardiology, Rhythmology, Angiology and Intensive Care Medicine EVK Dusseldorf and reports from the Division of Experimental Anesthesiology from the University Hospital Ulm.

The retrospective data analysis was approved by the local ethics committee of the Heinrich-Heine University Düsseldorf (Study-Number: 2021-1698).

Centers for Disease Control and Prevention (CDC). Advisory Committee on Immunization Practices (ACIP). Coronavirus disease 2019 (COVID-19) vaccines. Accessed July 6, 2021. https://www.cdc.gov/vaccines/acip/meetings/slides-2021-06.html. 

## Results

Specific patient data are described below. A summary of the data is provided in Table 1.

**Table 1:** Case description of the eight patients who developed cardiac side effects after vaccination against SARS-CoV-2

Case	Age (years)	Sex	Vaccine	Comorbidity	First/second injection	Time onset of the cardiac reaction (days)	Treatment	Relief of symptoms (days)
1	13	Male	Comirnaty©, BNT126b2	No	Second	3	Observation, intermediate care	5
2	28	Male	Comirnaty©, BNT126b2	No	Second	2	Observation	7
3	28	Male	Spikevax©, mRNA-1273	Diabetes mellitus type 1	First	2	Observation intermediate care	3
4	56	Female	Spikevax©, mRNA-1273	Metabolic syndrome	Second	2	Observation	7
5	42	Male	Comirnaty©, BNT126b2	No	Second	2	Observation	7
6	42	Male	Spikevax©, mRNA-1273	No	Second	10	Observation intermediate care	3
7	29	Female	Comirnaty©, BNT126b2	No	First	1	Observation intermediate care	5
8	15	Female	Comirnaty©, BNT126b2	girdle muscle dystrophy type 2d	Second	3	Hospitalization	5

### Patient 1

A 13-year-old healthy Caucasian male presented with chest pain 2 days after his second dose of SARS-CoV-2 mRNA-vaccine BNT162b2, Comirnaty©. No other symptoms were present. Troponin I level was elevated up to 150 ng/ml (normal level < 14 ng/ml) at the day of presentation and increased to 307 ng/ml on day 3 after admission. N-terminal fragment of the BNP precursor (NT-proBNP) was elevated to 520 pg/ml (normal level 10–157 pg/ml) on day 3. Further virological and microbiological examination revealed positive IgM-antibodies against *Mycoplasma pneumoniae* IgM EIA 0.97 (normal level < 0.80). Antibody titers against other cardiotropic infective agents such as *Toxoplasma gondii* or *Borrelia burgdorferi* were negative. Due to the inability to exclude *M. pneumonia* induced myocarditis therapy with azithromycin was initiated and continued for 4 days. A cardiac MRI was performed at day 2 detecting signs of a mild myocarditis with edema. The troponin I decreased rapidly to the normal range within 5 days. After 1 week in the intermediate care unit, the patient was discharged home fully recovered.

### Patient 2

**Fig. 2:**
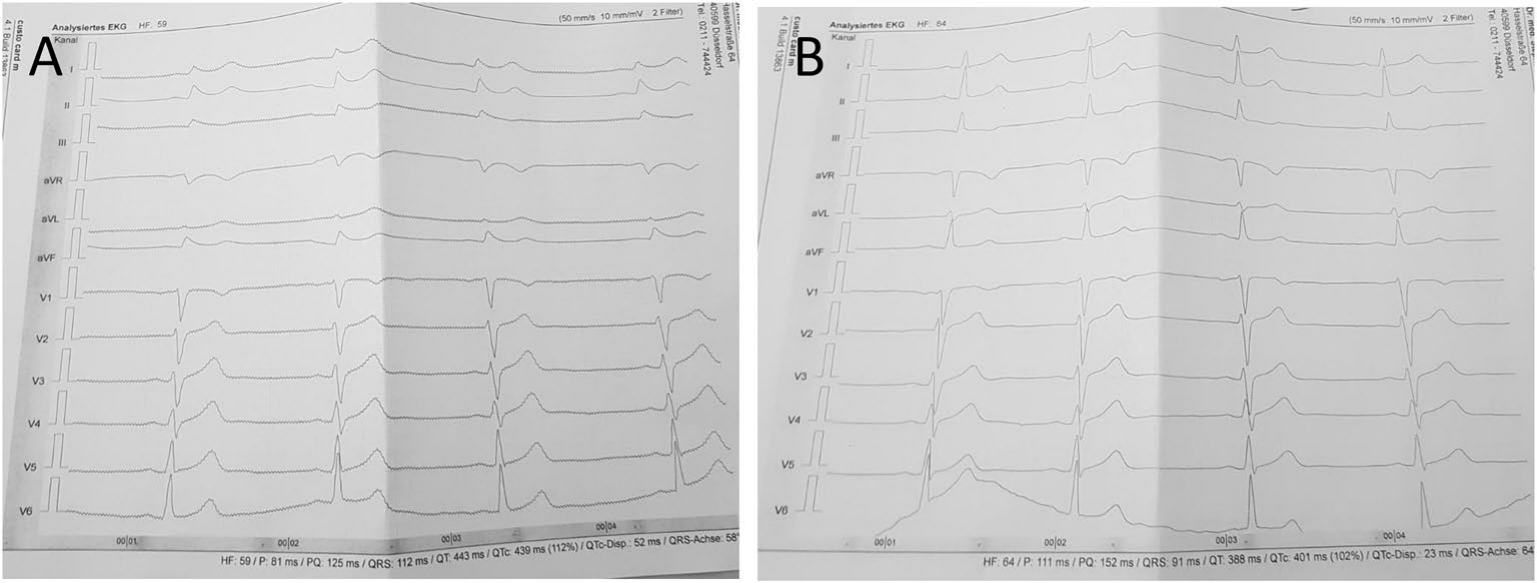
ECG performed on day 4 (**A**) and on day 6 (**B**) after the SARS-CoV-2 vaccination in a 28-year-old male. **A** Widening of the QRS complex (112 ms) in all ECG leads, and ST-Elevations in I, II, III, aVF and inverted in aVR. **B** Transient widening of the QRS complex has receded. There is left only a mild preterminal T negativity in lead III

A 28-year-old healthy caucasian male presented with dyspnea and left sided chest pain 3 days after the second dose of the mRNA-vaccine BNT162b2, Comirnaty©. A few hours after the second dose, the patient developed increasing dyspnea and angina with minimal exertion. On day 3, he resumed his exercise routine but presented to our outpatient clinic for evaluation. 4 days after the second dose, his symptoms persisted, but the troponin I and NT-proBNP, C-reactive protein and white blood cell count remained within normal limits. Electrocardiogram showed transient mild but significant ST elevations and widening of the QRS complex in the limb leads (Fig. 2). 6 days after the second vaccination dose, the transient ST alterations disappeared but a mild preterminal T-negativity in lead III persisted. NT-proBNP and Troponin I peaked at day 4, even though remained within normal limits, and decreased by day 6. It was higher on day 4, but still in the normal range. Transthoracic echocardiography was performed on post vaccination day 6, revealing neither right or left ventricular nor valvular abnormalities, pericardial effusion or wall motions dysfunction. No cardiac catheterization or cardiac magnetic resonance imaging were performed.. The symptoms fully resolved within 7 days.

### Patient 3

**Fig. 3:**
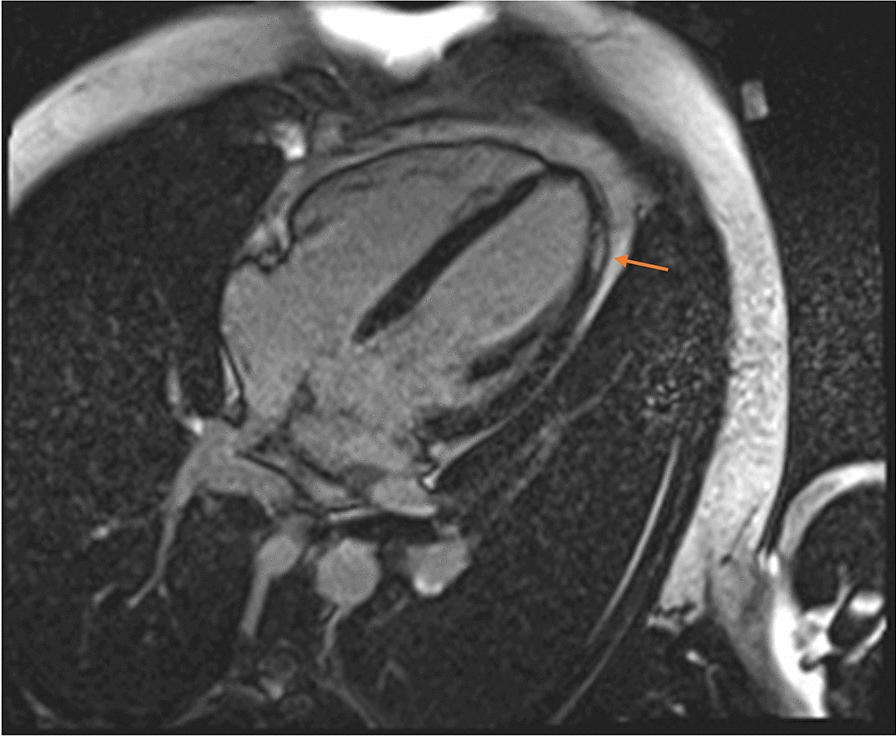
Cardiac magnetic resonance imaging (CMR) showed epicardial late enhancement apical and lateral suggestive of myocarditis. One week post vaccination, the angina was resolved, there were no arrhythmias on Holter monitor, and CK and Troponin returned within normal range. He was discharged on day 7 fully recovered

A 28-year-old male presented in the emergency Department 2 days after the second dose vaccination with mRNA1273, Spikevax© with acute chest pain and ST-elevation mimicking ST elevation myocardial infarction (STEMI). Due to a history of type-1-diabetes, an immediate cardiac catheterization was performed, ruling out obstructive coronary artery disease (CAD). Initial Troponin-T was 755 pg/ml (normal value < 14 pg/ml), rising to 894 pg/ml the next day; creatine kinase was 657 U/l, CK-MB 49 U/l, falling to 533 and 39 U/l, respectively. C-reactive protein was 4.0 mg/dl (normal value 0.8–1 mg/dl), reaching normal values on day 3. Initial echocardiography revealed slightly impaired left ventricular function; cardiac magnetic resonance (CMR) at 3 days (Fig. 3) showed a normalization of the left ventricular function and myocardial late enhancement.

### Patient 4

A 56-year-old female with a congenital metabolic disorder (lipoproteinemia) presented 2 days after her second dose of mRNA1273 Spikevax© with shortness of breath, sinus tachycardia with up to 100 beats per minute and chest pain. An ECG showed an inverted T-waves in the lateral leads. After one week, cardiac symptoms and T-wave inversion resolved, leaving the patient with some mild fatigue. No cardiac catheterization, magnetic resonance imaging or echocardiography were performed.

### Patient 5

A 42-year-old athletic male developed shortness of breath and sinus tachycardia with 110 beats per minute for the first time 2 days after his second dose with Comirnaty©. He did not have a known history of either cardiac disease or hypertension. An ECG revealed inverted T-waves in the lateral leads, with no changes in blood pressure. Climbing steps was difficult for several days. After one week, the cardiac symptoms disappeared. No cardiac catheterization, magnetic resonance imaging or echocardiography were performed.

### Patient 6

**Fig. 4:**
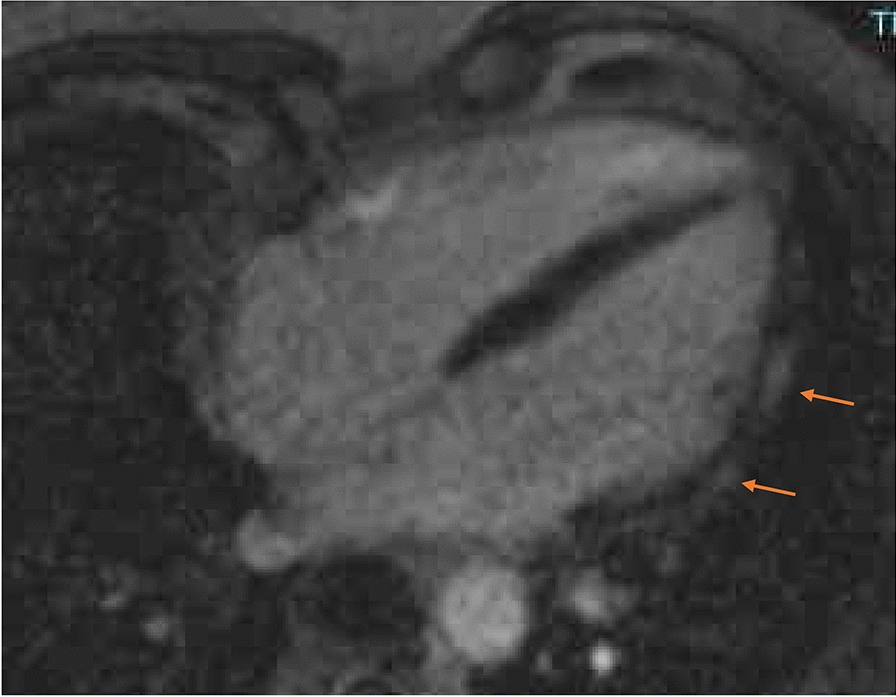
Cardiac magnetic resonance imaging (CMR) showed epicardial enhancement, predominantly in the lateral wall of the left ventricel as a sign of myocarditis

A 42-year-old male presented in the emergency department of the University of Düsseldorf with a single episode of acute chest pain 10 days after the second dose of the Spikevax^®^ vaccine. Immediately after vaccination he experienced fever and illness for a few days. Initial Troponin-T was 286 pg/ml (normal value < 14 pg/ml), falling to 92 pg/ml after 3 days from discharge. Creatine kinase was normal at admission and during the hospital stay. Coronary angiography excluded obstructive coronary  (CAD). MRI (Fig. 4) showed late enhancement indicating myocarditis (epicardial enhancement, predominantly in the lateral left ventricel).

### Patient 7

**Fig. 5:**
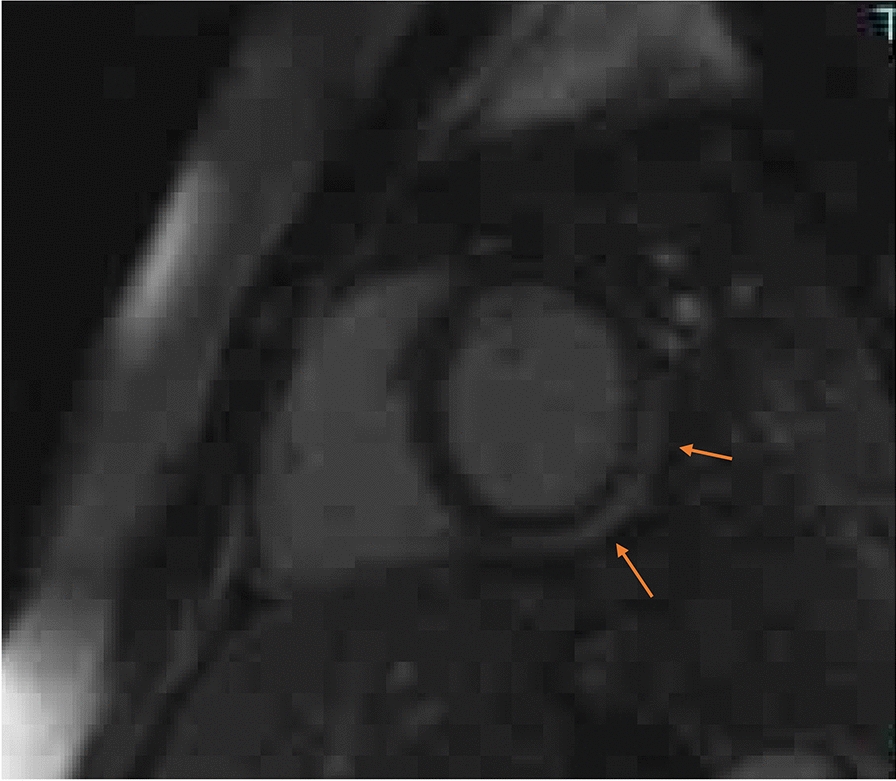
Cardiac magnetic resonance imaging (CMR) showed epicardial enhancement of the posterolateral LV wall as a sign of a myocarditis

A 29-year-old female presented in the emergency department with intermittent chest pain one day after the first dose of the BNT126b, Comirnaty© vaccine. Initial Troponin-T was 414 pg/ml (normal value < 14 pg/ml), rising up to 440 pg/ml and falling to 56 pg/ml after 4 days. Creatine kinase was 484U/l and normalized at discharge after 5 days. The cardiac MRI showed signs of myocarditis, epicardial enhancement of the posterolateral LV wall (Fig. 5).

### Patient 8

A 15-year-old caucasian female patient with a known limb girdle muscle dystrophy type 2D presented with chest pain 3 days after the second dose of SARS-CoV-2 mRNA-vaccine Comirnaty©. The day after vaccination, she developed fever up to 39 °C, reporting similar symptoms after the first dose. Due to progressive chest pain, she was admitted to the pediatric ward. Troponin T levels were elevated up to 1438 ng/ml at admission, while NT-proBNP was elevated to 300 pg/ml (normal level 6–158 pg/ml). Both Troponin T and NT-proBNP decreased rapidly during the following days, reaching nearly normal levels at discharge (Troponin T 81 ng/ml, NT-proBNP 62 pg/ml).

Because of the muscular dystrophy, cardiac enzymes were differentiated into Troponin I (264 ng/ml, norm < 17.5 ng/ml) and Troponin T (136 pg/ml, norm < 14 pg(ml).

A cardiac magnetic resonance imaging (CMR) was performed on day 4 revealing signs of mild myocarditis with edema and elevated T2-relaxation-times. Echocardiography showed decreased strain function which normalized within the next 2 days.

Further virological and microbiological examination did not reveal an infectious myocarditis.

After 5 days in the intermediate care unit, the patient was discharged without any residual symptoms.

#### Discussion

Our small case series of eight patients highlights the potential link between BNT126b2 or mRNA-1273 vaccination against SARS-CoV-2 and myocarditis or pericarditis after a short time period from the second dose, in young and healthy individuals. This is consistent with data in the literature [13].

Our population was young (age range between 13 and 56 years), with no pre-vaccination cardiac history. They presented at our institution with dyspnea or angina as their main complaint. In most cases, the disease appeared within 2 to 3 days after the second dose and was accompanied by changes in the ECG, echocardiography, or MRI of the heart; some patients showed signs of myo- or pericarditis. In every patient, there was a complete resolution of the symptoms within 5–7 days under supportive treatment, allowing discharge at home with no long-term sequelae.

Myocarditis is a rare post-vaccination side effect, recently observed 1–5 days after the administration of mRNA-based COVID-19 vaccines, such as BNT126b2 or mRNA-1273. The onset and the symptomatology are consistent with the data reported by Diaz et al. in JAMA [35], Das et al. in Children [12] and Jain et al. in Pediatrics [36].

**Fig. 6:**
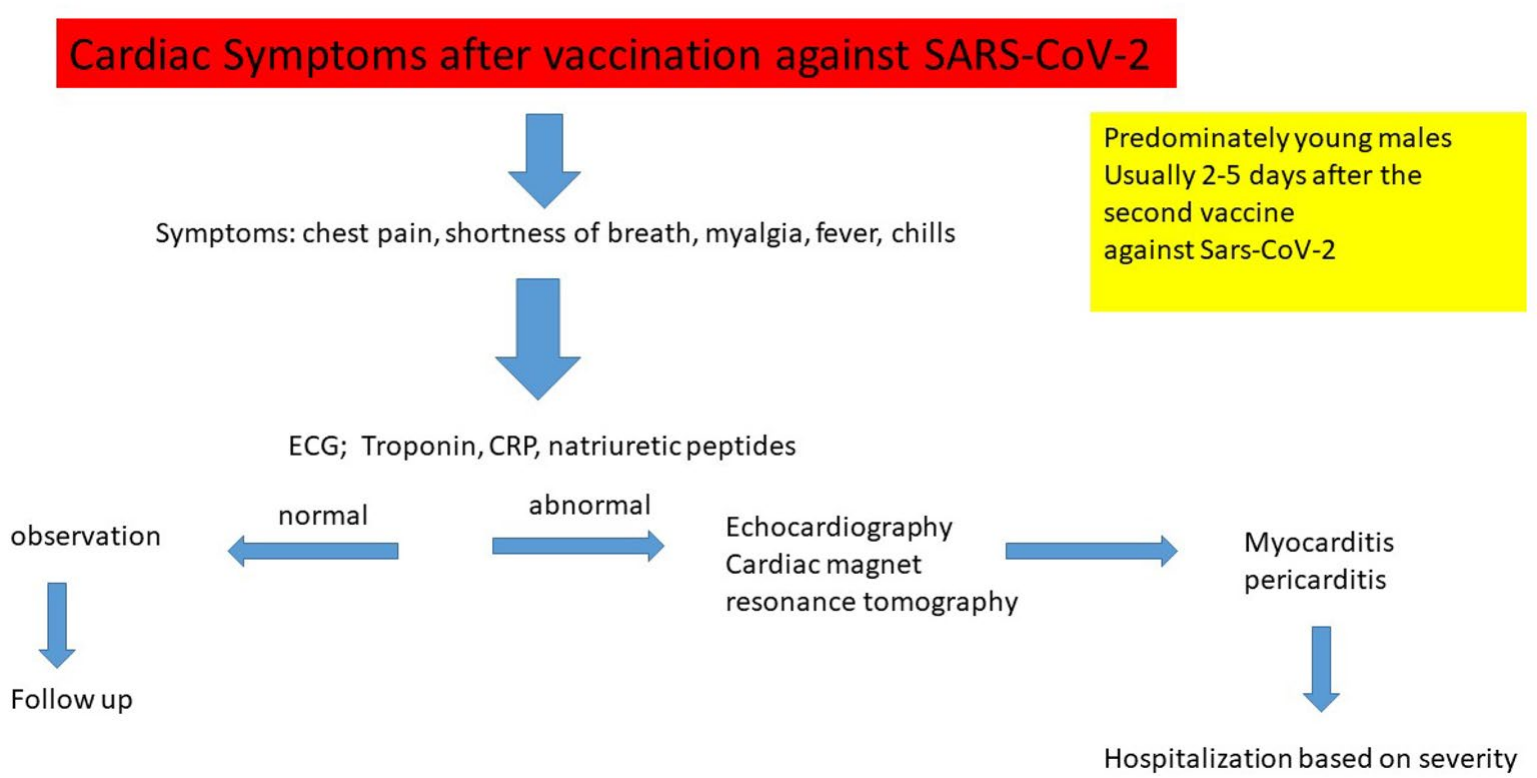
Flowsheet for diagnostic work up once cardiac events occurred after SARS-CoV-2 vaccination

The pathophysiology of post vaccination myocarditis still remains unclear. The final diagnosis of myocarditis or pericarditis and the demonstration of a causal relationship to the vaccination is challenging. The CDC defined chest pain, dyspnea, feelings of palpitations, fluttering or pounding heart as signs and symptoms for possible myocarditis or pericarditis. Diagnostic work-up in includes ECG, Troponin, CK/CK-MB, CRP and echocardiography (Fig. 6).

Myocardial biopsy is still considered the gold standard for diagnosis but is not routinely performed due to its invasiveness.

Our patient population is quite heterogeneous as far as age, preexisting conditions, and test abnormalities, suggesting that a high level of suspicion is needed when taking the history of patients complaining of angina in the immediate postvaccination period.

Patient 1 is an outlier, due to the copresence of M.pneumoniae-IgM. *M. Pneumoniae* can present with angina, which responds to the proper antibiotic treatment. The proper diagnosis in this case is paramount to provide the correct treatment. However, the sensitivity of serological tests against *M pneumonia* at the symptom onset is only 31.1% and IgM can be detected up to 1 year from the infection. In this case, mycoplasma myocarditis was excluded due to the lack of other respiratory symptoms expected during an active infection.

Post vaccination myocarditis and pericarditis have been described in the literature. The reported incidence is 1:20.000 after the second dose, affecting mostly younger men. The phenomenon is transient and typically resolves within 3–5 days, frequently without any treatment other than supportive measures and rest. Healthcare professionals should have a high index of suspicion to make the proper diagnosis. Vaccinated individuals should seek medical attention when experiencing new onset of chest pain, shortness of breath, palpitations or arrhythmias after receiving mRNA based COVID-19 vaccines.

## Conclusions

Cardiac side effects such as myocarditis are a rare side effect that can present as a localized, transient event several days after the first or second dose of the mRNA-based COVID-19 vaccines. Vaccinated patients should be notified that myocarditis could occur especially in young males. Further investigations on the precise molecular and cellular mechanisms underlying this side effect are needed to understand why and when rare adverse events may occur after S protein encoding vaccines. Close clinical follow-up of confirmed cases should be performed. Affected patients should consider cardiology follow-up.

## Data Availability

All data and materials can be accessed via CM and MK.

## Abbreviations

CDC: Centers for disease control and prevention
COVID-19: Coronavirus disease 2019
CK: Creatine kinase
CRP: C-reactive protein
DNA: Deoxyribonucleic acid
IgM: Immunoglobulin M
mRNA: Messenger ribonucleic acid
SARS-CoV-2: Severe acute respiratory syndrome coronavirus-2
MRI: Magnetic resonance imaging
NT-proBNP: N-terminal probrainnatriuretic peptide
STEMI: ST-segment elevation myocardial infarction
CK: Creatine kinase
CK-MB: Creatine kinase M: myocardial B: brain
LV: left ventricular
CAD: coronary artery disease
